# Simultaneous Laparoscopic Ventral Hernia Repair and Peritoneal Dialysis Catheter Placement in Patients With Chronic Renal Failure: A Single-Center Retrospective Analysis

**DOI:** 10.7759/cureus.83521

**Published:** 2025-05-05

**Authors:** Gianpaolo Marte, Giuseppe Surfaro, Gennaro Argentino, Andrea Camocardi, Francesco Guida, Raffaele Genualdo, Mariano F Armellino

**Affiliations:** 1 Department of General Surgery, Ospedale del Mare, Naples, ITA; 2 Department of Nephrology and Dialysis, Ospedale del Mare, Naples, ITA

**Keywords:** abdomen ventral hernia, end stage renal disease (esrd), intraperitoneal onlay mesh (ipom), laparoscopic ventral hernia repair, mesh repair, peritoneal dialysis catheter, peritoneal dialysis complication, tenckhoff catheter, ventral and incisional hernia

## Abstract

Background

In patients undergoing peritoneal dialysis (PD), there is a high incidence of ventral hernia during the first five years of follow-up. Early diagnosis of the occurrence of ventral hernias in PD patients is very important to ensure their surgical treatment as quickly as possible.

Methods

A retrospective analysis of prospectively collected data was conducted between January 2022 and May 2023. All patients who have undergone peritoneal catheter implantation by laparoscopy and concomitant ventral hernia repair according to the laparoscopic intra-peritoneal onlay mesh technique were enrolled. Primary outcomes include operative time, blood loss volume, conversion to open surgery, hospital stay, hematoma, chronic pain, complication rate according to Clavien Dindo score, hernia recurrences, and peritoneal catheter displacement or malfunction.

Results

The mean operative time was 40 minutes (range: 30-60 minutes). Intraoperative blood loss was less than 30 ml. No conversion to laparotomy was needed. Considering a mean follow-up of six months, no Clavien Dindo grade > 2 was recorded. One hematoma appeared on postoperative day 4. No chronic pain, defined as pain lasting >3 months, was recorded. No hernia recurrence was registered, but one was an asymptomatic bulging Early initiation of PD was achieved, with no reported malfunctions of the peritoneal catheter or infusion system to date.

Conclusion

For patients on PD with ventral hernia, laparoscopic intraperitoneal onlay mesh (IPOM)* *repair has the advantages of less trauma, simultaneous treatment of occult hernias, adjustment and fixation of PD tubes, low incision complication rates, and low recurrence rates. IPOM repair can be performed safely and effectively in this population group; thus, it is a procedure worth promoting.

## Introduction

Chronic renal failure (CRF) is a progressive and irreversible decline in renal function, leading to impaired glomerular filtration rate. Among the treatment options, peritoneal dialysis (PD), introduced in 1946 [1], has proven to be an effective and efficient renal replacement therapy. PD offers several advantages over hemodialysis (HD), including the preservation of residual renal function, independence from dialysis centers, the ability to maintain a normal social life, remote monitoring, lower costs, and rapid isolation during pandemics like COVID-19 [2,3].

In patients undergoing PD, the incidence of hernias reaches approximately 50% within five years of follow-up. This high prevalence is attributed to increased intra-abdominal pressure and tissue scarring after surgery [4,5]. The success of PD is strongly dependent on maintaining the integrity of the abdominal wall, making early detection and management of hernias crucial in preventing PD failure and avoiding conversion to HD. Studies have shown that PD patients are at higher risk for ventral hernias compared to the general population, with risk factors including longer PD duration, polycystic kidney disease, dialysis solution volume, elevated body mass index (BMI), and prior abdominal surgeries.

Early diagnosis and timely surgical intervention are essential to prevent complications such as hernia incarceration, strangulation, genital edema, dialysis fluid leakage, and inefficiency or failure of PD. While mesh repair via open surgery is a standard treatment for ventral hernias, concerns remain about mesh infections leading to exposure, erosion, or recurrence [6].

Most surgical repairs for hernias in PD patients are performed using open techniques, with limited studies exploring laparoscopic approaches. Laparoscopic repair techniques, such as trans-abdominal pre-peritoneal (TAPP) or totally extra-peritoneal (TEP) repairs for inguinal hernias, raise concerns about peritoneal fibrosis and reduced PD efficiency. For other ventral hernias requiring laparoscopic intra-peritoneal dual mesh placement, risks include adhesion formation and potential mesh infections during PD-related peritonitis [7].

The most common ventral hernias in PD patients are inguinal, umbilical, femoral, peri-catheter, and incisional hernias, with the latter being particularly prevalent in individuals with a history of abdominal surgeries. The advent of laparoscopic techniques has revolutionized hernia repair, offering faster recovery, fewer complications, and improved quality of life. However, in patients with CRF or those who require continuous PD, laparoscopic repair is still debated due to concerns about peritoneal catheter infections [8].

PD catheter placement, first developed in 1968, is a critical component of successful PD. Historically, catheters were inserted through a small laparotomy with blind placement, leading to high obstruction rates (up to 36%). Alternative techniques using fluoroscopy, peritoneoscopy, and laparoscopy have since been developed, although consensus on the optimal approach remains elusive [9].

While some studies highlight the benefits of laparoscopic insertion, others suggest similar outcomes in terms of complications and catheter survival between laparoscopic and open techniques. Despite varying opinions and evidence, laparoscopic catheter insertion is increasingly recognized as a reliable method for peritoneal access [10,11].

The aim of this paper is to present a single-step, concurrent laparoscopic technique combining ventral hernia repair with mesh implants and Tenckhoff catheter placement. We report preclinical results from a single-center case series, focusing on the effectiveness and safety of this approach.

## Materials and methods

A retrospective analysis of prospectively collected data was conducted between January 2022 and May 2023. The study included patients from the Nephrology Department at Ospedale del Mare, Naples, Italy, enrolled by GA, a nephrologist with over 16 years of experience in PD.

Inclusion criteria included patients who underwent laparoscopic peritoneal catheter implantation and simultaneous ventral hernia repair using the intraperitoneal onlay mesh (IPOM) technique.

The hospital protocol for PD candidates involves a preoperative surgical consultation to assess abdominal wall integrity, particularly in patients with a history of abdominal surgery. Ventral hernias were diagnosed through physical examination (standing and supine positions) and imaging (ultrasound or CT), particularly in obese patients where physical examination was inconclusive.

The primary causes of renal failure in the cohort were diabetic nephropathy (n=3) and hypertensive nephropathy (n=2). All patients had at least three preoperative comorbidities, assessed using the Charlson Comorbidity Index (CCI) [12].

Demographic and clinical data collected included age, sex, BMI, number of prior intra-abdominal surgeries, presence of chronic obstructive pulmonary disease (COPD), diabetes mellitus, hypertension, cardiovascular disease, and type of hernia (classified per exertional heatstroke (EHS)) [13].

Surgical approach

Antiplatelet therapy was discontinued seven days preoperatively, and oral anticoagulants were replaced with low molecular weight heparin for the same period. Postoperatively, patients were fasted until bowel transit resumed, with parenteral rehydration and local compression applied to prevent complications.

Outcomes measured included operative time, blood loss, conversion to laparotomy, hospital stay, hematoma, chronic pain, complications (graded using Clavien-Dindo [14]), hernia recurrence, and peritoneal catheter malfunction or displacement.

Of the five patients, two presented with primary umbilical hernias, two with incisional hernias, and one with both umbilical and incisional hernias; all were managed laparoscopically using IPOM [15]. All procedures were performed by a single expert laparoscopic surgeon (GS), with Gore Synecor meshes implanted. The mean follow-up period was 12 months, during which abdominal ultrasonography was performed in cases of local complications or suspected hernia recurrence.

Demographic data are summarized in Table 1.

**Table 1:** Patient characteristics BMI: body mass index; CRF: chronic renal failure; COPD: chronic obstructive pulmonary disease; CVD: cardiovascular disease

Patients	Sex	Age (years)	BMI	CRF (cause)	COPD	CVD	N. prior surgery	Preoperative dialysis (day)	Primary hernia	Incisional hernia	EHS
1	F	63	33.3	Diabetic nephropathy	No	Yes	1	15 days	Umbilical hernia		M3W2R0
2	M	76	26.2	Diabetic nephropathy	No	Yes	3	10 days	Umbilical hernia	Incisional hernia	M3W1R0
3	M	74	30.1	Hypertensive nephropathy	Yes	Yes	No	7 days	Umbilical hernia		M3W1R0
4	F	88	19.5	Hypertensive nephropathy	Yes	Yes	1	21 days		Incisional hernia	M2/3W2R1
5	M	77	24.5	Diabetic nephropathy	Yes	Yes	1	15 days		Incisional hernia	M3W1R0

## Results

The mean operative time was 40 minutes (range: 30-60 minutes), with intraoperative blood loss consistently less than 30 mL. No conversions to laparotomy were required. Over a mean follow-up period of six months, no complications greater than Clavien-Dindo grade II were recorded.

Two patients developed a hematoma on postoperative day 4, which was successfully managed with medical therapy. No cases of chronic pain (defined as pain lasting more than three months) were reported.

PD was initiated early in all patients, with no malfunctions of the catheter or infusion system observed. None of the patients required temporary transfer to HD, and no discontinuation of PD due to parietal defects occurred.

At the 12-month follow-up, no hernia recurrence was detected, and one asymptomatic bulging was registered; however, reintervention was not necessary at the time of assessment. Surgical results are reported in Table 2.

**Table 2:** Surgical outcomes CP: chronic pain; CD: Clavien-Dindo complication classification

Patients	Operative time (minutes)	Blood loss (ml)	Conversion	Hospital stay (days)	Hematoma	CP	CD score	Hernia recurrence	Catheter malfunction
1	40	20	No	5	No	No	I	No	No
2	30	20	No	7	Yes	No	0	No	No
3	35	10	No	3	No	No	0	No	No
4	60	25	No	7	Yes	No	II	No (bulging)	No
5	35	30	No	7	No	No	I	No	No

Patients were discharged between postoperative days 3 and 7. Peritoneal lavage began on the second postoperative day, with sessions twice weekly during the first week using 200 mL of dialysate per session. The volume and frequency of exchanges were progressively increased over the following three weeks. Two patients required early dialysis ("early break-in") after 10 and 14 days due to dialysis needs, with no associated complications.

Complications

One patient experienced minor dialysate leakage following hernioplasty, which resolved within a few days after reducing the dialysate load volumes. Continuous ambulatory PD (CAPD) was maintained without interruptions. No further leakage was observed during the follow-up period.

None of the patients developed peritonitis, either in the perioperative period or throughout the entire follow-up period.

Dialysis outcomes

All patients achieved improved dialysis quality, with no issues related to purification or adherence to prescribed therapy. At the time of this report, all patients remained on PD and continued to adhere to their treatment regimens without significant complications. Nephrology outcomes are all listed in Table 3.

**Table 3:** Nephrology outcomes

Patients	Catheter malfunction	Leakage of dialysate	Mesh infection	Wound infection	Leakage of dialysate	Peritonitis	Infection exit
1	No	0	0	0	0	0	0
2	No	0	0	0	0	0	0
3	No	0	0	0	1	0	1
4	No	0	0	0	0	0	0
5	No	0	0	0	0	0	0

## Discussion

Reducing the incidence of parietal defects in patients undergoing PD is a shared priority for nephrologists and surgeons. Thorough pre-PD evaluations - including clinical examinations for ventral parietal defects and laparoscopic catheter placement, allowing direct visualization of the peritoneal cavity - play critical roles in achieving this goal [16].

Surgery during PD for symptomatic hernias diagnosed before the start of PD is generally not recommended because of the risks involved. These include compromised PD efficiency (e.g., sequestration of dialysate fluid in the hernia sac) and complications from the parietal defect itself, such as incarceration, strangulation, or the formation of intraperitoneal adhesions. This approach should be reserved only for patients with high anesthetic risk (American Society of Anesthesiologists (ASA) score > 3) [7].

Several studies and guidelines (e.g., Society of American Gastrointestinal and Endoscopic Surgeons (SAGES), International Society for Peritoneal Dialysis (ISPD)) advocate for the establishment of multidisciplinary "Access Teams" that include surgeons, nephrologists, anesthesiologists, and specialized nurses. These teams develop local protocols and provide training to ensure prompt access to care, supervision, and management during both catheter placement and subsequent surgical interventions [17,18].

In our cohort, all peritoneal catheters were placed laparoscopically, with five patients undergoing simultaneous ventral hernia repair. This practice aligns with recommendations from ISPD and SAGES guidelines, which support concurrent hernia repair during PD catheter placement to minimize complications and optimize outcomes [18,19].

The prevalence of pre-existing hernias in PD patients is reported between 5.5% and 15% [20-22], with ventral hernias being the most common type. Umbilical hernias are particularly prevalent among PD patients [23,24]. Our results are consistent with these trends and show that simultaneous laparoscopic ventral hernia repair with intra-peritoneal mesh placement and PD catheter insertion is both safe and effective. This is supported by the absence of postoperative dialysate leaks, catheter infections, or hernia recurrences in most cases during follow-up.

To the best of our knowledge, this is the first Western report on this combined laparoscopic approach using intra-peritoneal mesh. A recent study from Asia similarly demonstrated promising results with combined procedures, albeit with a smaller cohort and a different hernia distribution (primarily inguinal hernias) [25]. Our outcomes are consistent with the literature, including reports by Garcıa-Urena et al. and Sodo et al., who documented low complication rates and favorable long-term outcomes in combined procedures using mesh [6,22]. Furthermore, a recent National Surgical Quality Improvement Program (NSQIP) analysis [19] of 330 combined procedures showed no increase in 30-day morbidity or mortality compared to PD catheter placement alone.

Contrasting reports, such as a 2021 study suggesting increased morbidity with combined procedures [25], highlight the need for further investigation. However, the lack of detail regarding the hernia repair techniques and meshes used in that study limits direct comparisons with our findings.

Our study has some limitations. As a single-center retrospective analysis with a small cohort and without a control group, its generalizability is constrained. However, the consistent application of advanced laparoscopic techniques and the utilization of modern prosthetic materials further strengthen the validity of our conclusions. While laparoscopic hernia repair is now the standard of care for the general population, the lack of specific guidelines for PD patients highlights the need for further research to standardize practices in this subset. Advances in prosthetic materials for minimally invasive hernia repair may pave the way for broader adoption of laparoscopic techniques in PD patients [26,27].

Future directions

The preoperative diagnosis of ventral hernias in patients scheduled for PD catheter placement is crucial to reducing the incidence of parietal defect-related complications during PD. Identifying and addressing hernias prior to initiating PD enhances patient outcomes by minimizing risks such as hernia-related complications and PD inefficiency.

Laparoscopic IPOM repair offers several advantages in PD patients with ventral hernias. These include reduced surgical trauma, the ability to address occult hernias simultaneously, optimal adjustment and fixation of PD catheters, low rates of incision-related complications, and a low risk of hernia recurrence. Based on our findings, IPOM repair is a safe and effective option for this patient population and deserves further consideration as a preferred technique in similar clinical settings.

The current study is limited by its retrospective design, small cohort size, and lack of a control group. To validate these findings and establish evidence-based guidelines, prospective studies with larger sample sizes, longer follow-up periods, and comparative analysis against other surgical techniques are needed.

## Conclusions

Despite study limitations, our data provide valuable insights into the feasibility and safety of laparoscopic IPOM repair with simultaneous PD catheter placement. These results challenge the long-standing belief that intra-peritoneal mesh placement is contraindicated in patients undergoing PD. With advancements in prosthetic materials and minimally invasive surgical techniques, this approach has the potential to become a standard practice for managing ventral hernias in PD patients. A well-designed, randomized controlled trial would help solidify these preliminary findings and pave the way for broader adoption of this technique.
